# A Blood Pressure Monitoring Method for Stroke Management

**DOI:** 10.1155/2014/571623

**Published:** 2014-08-17

**Authors:** Heather Ting Ma

**Affiliations:** Department of Electronic and Information Engineering, Harbin Institute of Technology Shenzhen Graduate School, Shenzhen 518055, China

## Abstract

Blood pressure is one important risk factor for stroke prognosis. Therefore, continuous monitoring of blood pressure is crucial for preventing and predicting stroke. However, current blood pressure devices are mainly air-cuff based, which only can provide measurements intermittently. This study proposed a new blood pressure estimation method based on the pulse transit time to realize continuous monitoring. The proposed method integrated a linear model with a compensation algorithm. A calibration method was further developed to guarantee that the model was personalized for individuals. Variation and variability of pulse transit time were introduced to construct the compensation algorithm in the model. The proposed method was validated by the data collected from 30 healthy subjects, aged from 23 to 25 years old. By comparing the estimated value to the measurement from an oscillometry, the result showed that the mean error of the estimated blood pressure was −0.2 ± 2.4 mmHg and 0.5 ± 3.9 mmHg for systolic and diastolic blood pressure, respectively. In addition, the estimation performance of the proposed model is better than the linear model, especially for the diastolic blood pressure. The results indicate that the proposed method has promising potential to realize continuous blood pressure measurement.

## 1. Introduction

It has been reported that ambulatory blood pressure values were linearly related to stroke risk, which has stronger predictive power than screening blood pressure [1]. The predictive value of home blood pressure measurement increased progressively with the number of measurements within 24 hours [2]. Studies also showed that hypertension would increase the risk of stroke [3], especially for those subjects who had stroke history [4]. It was found that a 10 mmHg greater reduction in systolic blood pressure (SBP) would be associated with a 31% reduction in stroke risk within a follow-up duration of average 4.5 years for the elderly [5, 6]. Therefore, continuous blood pressure monitoring is crucial for both predicting stroke and hypertension management [7, 8]. 24-hour ambulatory blood pressure monitoring has been increasingly used in clinic for hypertension management [9–11]. Current blood pressure devices (by oscillometry or sphygmomanometery) are mainly based on air-cuff, which only can measure blood pressure intermittently and may not be suitable for long term blood pressure monitoring. Therefore, cuffless blood pressure monitoring method would be valuable in stroke prevention and management.

Pulse transit time (PTT) has been reported to be correlated with blood pressure, especially for the SBP [12–17], and has been proposed as a potential surrogate of blood pressure [18–20]. Pulse transit time can be measured between the characteristic points of the electrocardiography (ECG) and photoplethysmography (PPG) at peripheral sites [17, 21]. Since ECG and PPG measurements can be implemented by wearable devices, PTT provides a very practical solution for continuous blood pressure monitoring. A lot of studies have focused on the blood pressure estimation by using PTT [22–26] and different applications have been proposed based on the blood pressure estimation methods [27–29]. Linear model [17] was mostly adopted to describe the relationship between blood pressure and PTT. But the linear model cannot provide accurate estimation because PTT was found highly correlated with SBP rather than diastolic blood pressure (DBP) [30]. An accurate model describing the relationship between PTT and blood pressure is crucial for the PTT-based blood pressure estimation. Sophisticated models were further proposed to enhance the accuracy of PTT-based blood pressure estimation. Some studies investigated the relationship between PTT and blood pressure under static and exercise status [26, 31, 32]. Considering the relationship between PTT and blood pressure could vary from person to person, calibration was proposed by some researchers to design personalized estimation model [33, 34]. Some studies took advantage of the hydrostatic pressure change in the calibration [18, 35, 36]. Nevertheless, the major challenge for PTT-based blood pressure measurement is to derive a personalized estimation model.

Apart from the estimation accuracy, implementation of the estimation model in a device for clinical or healthcare use is the ultimate goal. The linear mapping between PTT and blood pressure has not been proved to provide the best blood pressure estimation. However, it is still the best applicable for coarse blood pressure trend indications [37]. Therefore, in present study, we proposed a new method for PTT-based blood pressure estimation by integrating a compensation part in the linear model, which was further combined with a new calibration approach.

## 2. Methodology

A previous study pointed out that PTT variability had high coherence with heart rate variability and blood pressure variability [38], both of which reflect neural regulation of cardiovascular system. Douniama et al. [37] also suggested introducing variability information in PTT-based blood pressure estimation to reflect the frequency-dependent arterial vessel compliance and the autonomic nervous system on vascular tone. Therefore, in current study, the variation in PTT and PTT variability are regarded carrying important information of blood pressure regulation and are integrated into the traditional linear model as a compensation part. Further, a posture-based calibration method was adopted by using the hydrostatic pressure change to personalize the estimation model for individuals.

### 2.1. Subjects and Devices

This study involved 31 healthy subjects (aged from 23 to 25 years) without known cardiovascular abnormalities. The subjects were recruited in a university campus with submitting a written consent with full understanding of the experiment procedure. Each subject was asked to refrain from coffee and alcohol at least 2 hours and instructed about the procedures before conducting the experiment. Then the experiment was carried out in a temperature-controlled room (24 ± 2°C) for all the subjects.

For the data collection, standard lead I ECG and reflective PPG signals were recorded simultaneously by a self-designed device at a sampling rate of 250 Hz for each channel and digitized by a 12-bit A/D converter. The ECG signal was collected from the index fingertip of left hand and the index and middle fingertips of right hand, while the PPG signal was collected from the index fingertip of right hand at the same time. Standard blood pressure was measured by an oscillometry (OMRON HEM-7012, Japan) at subjects' left upper arms.

### 2.2. Experimental Protocol

The experiment procedure was arranged into four sessions, namely, pretest, calibration sitting, calibration standing, and estimation test. In pretest session, each subject was required to sit down and relax for 10 minutes to stabilize his/her blood pressure. During this time, the participant got prepared for the experiment, such as wearing the sensors. The rest of the three sessions followed a routine data collection procedure, which is shown in Table 1. Considering the blood pressure fluctuation would influence the calibration accuracy, the difference between the blood pressure measurements by the oscillometry in each session was checked. Specifically, for each routine data collection, if the difference between the first two blood pressure measurements was less than 5 mmHg, the first measurement was adopted as the blood pressure value for this dataset. If the difference ranged between 5 and 10 mmHg, another measurement would be carried out after 5-minute rest and the average of the three measurements would be taken as the blood pressure value for this dataset. If the difference exceeded 10 mmHg, the experiment would be ceased and another appointment would be made with the participant. The measurements by oscillometry in the same session were carried out under a peaceful situation so that the blood pressure was supposed to be stable. When the blood pressure measurements under the same conditions exceeded 10 mmHg, some unstable factor was supposed to happen during the measurement. The unstable factor could be a wrong operation of the experiment, motion artifact, or unstable physiological status, which would also influence the estimation results. Therefore, the data collected under such situation were discarded.

The experiment outline is summarized in Table 2. Between any two successive sessions, there was a rest with at least 5 minutes. Specifically, data collected in the middle two sessions were used for calibration and that in the last session was used for blood pressure estimation. In the two calibration sessions, the subject sat and stood upright during the data collection, respectively.

### 2.3. Parameter Extraction

**Figure 1:**
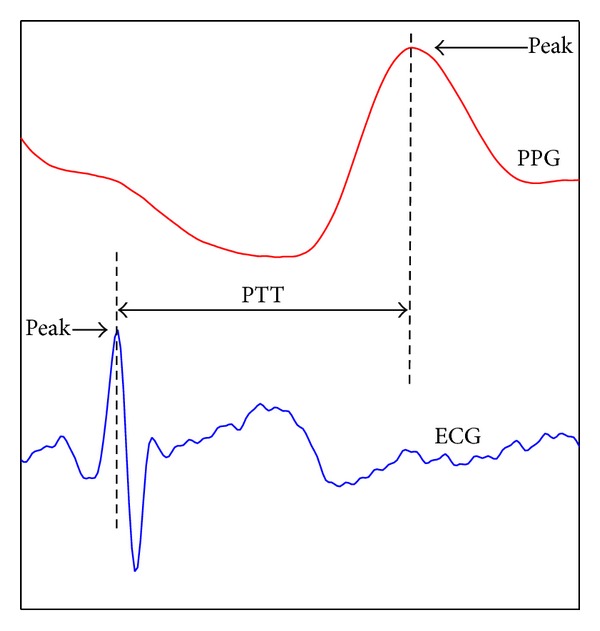
Pulse transit time is defined as the time interval between the R-peak of ECG and the peak of PPG within the same cardiac cycle.

Ninety-three datasets were finally recorded for the 31 subjects and processed offline. The raw data were first filtered by a sliding window with window length of 10 milliseconds. The beat-to-beat PTT was defined as the time interval between the R-wave of ECG and the peak of the PPG pulse within the same cardiac cycle (see in Figure 1). The fluctuation of the recorded signals was checked, where the signal with large fluctuation was considered as invalid because it might indicate an unstable physiology conditions during the recording. By such criteria, one subject's data were removed. Finally, 90 datasets from 30 subjects were included in the blood pressure estimation analysis.

### 2.4. Estimation Model

In previous study, it has been confirmed that the variation in PTT can reflect the blood pressure changes [17] based on which linear model was proposed for blood pressure estimation. However, no factor of blood pressure regulation mechanism has been included in the traditional linear model. Neural control is one important BP regulation mechanism. Studies have shown that vital cardiovascular parameters' variability can reflect the neural regulation [39–41]. Further, a previous study showed that the variability in blood pressure and PTT has high coherence [38]. Considering the feasibility of the model implementation, we chose to formulate the model based on the traditional linear algorithm and integrate with the variation and variability of PTT as the indication of neural control. Finally, a blood pressure estimation model was formulated as shown in the following equation:

(1) $$\begin{array}{c} \text{BP}=\frac{a}{\text{PTT}}+b+c*\text{VPTT}+d*\left(\text{PTTV}-\text{PTT}{\text{V}}_{0}\right), \end{array}$$

where BP refers to blood pressure value;* a*,* b*,* c*, and* d* are coefficient constants; PTT is the pulse transit time value for estimation; PTTV is the PTT variability during the signal recording, which is defined as (2); PTTV_0_ is PTTV at the* Calibration-sitting* session; and VPTT is the PTT variation at the measurement time, which is formulated as (3):

(2) $$\begin{array}{c} \text{PTTV}=\;\sqrt{\frac{\sum_{i=1}^{N}{\left(\Delta \text{PT}{\text{T}}_{i}-\text{mean}(\Delta \text{PTT})\right)}^{2}}{N-1}} \end{array}$$

(3) $$\begin{array}{c} \text{VPTT}=\;\frac{\text{PTT}-{\text{PTT}}_{0}}{{\text{PTT}}_{0}}, \end{array}$$

where ΔPTT is the difference between any two successive PTTs;* N* is the number of PTT used for variability calculation. In present study,* N* was set to 5. Due to the signal fluctuation, PTT value in (1) was set as the average of the 5 measurements. In other words, (1) can provide blood pressure estimation for each beat based on past 5 measurements.

### 2.5. Calibration

Considering that the relationship between blood pressure and PTT varies from subject to subject, individual calibration is necessary for blood pressure estimation. Specifically, the coefficient constants in (1) should be calibrated for the individual blood pressure estimation. First, all coefficient constants were derived by data regression by using all collected data from the subjects. Then coefficient constants *a* and *b* were further calibrated for each individual by calibration.

Blood pressure would vary due to hydrostatic effect, which provides an effective solution for the calibration [35, 42]. Different body postures, such as sitting and standing, will result in different blood pressure situation. Therefore, in current study, the scenario of personalized model calibration is to use the recorded PTT and blood pressure values at* Calibration-sitting* and* Calibration-standing *sessions to derive the value of *a* and *b* in (1). Thereafter, blood pressure estimation was carried out on the dataset measured in* Estimation-test* session.

## 3. Results

In order to evaluate the accuracy of the proposed method, the estimated blood pressure values were compared with the paired blood pressure measurements from the oscillometry. Further, we also compared the blood pressure value estimated from the proposed method with that by linear model, as shown in the following equation:

(4) $$\begin{array}{c} \text{BP}=\frac{a}{\text{PTT}}+b, \end{array}$$

where *a* and *b* are coefficient constants and also calibrated by the datasets from* Calibration-sitting* and* Calibration-standing* sessions. For model implementation, in order to be comparable with the proposed method, PTT value in (4) was also set as the average of the 5 measurements. As a result, the estimations from both the proposed method and the traditional linear model were based on 5 measurements of PTTs.

The correlation between any paired variables was analyzed by the correlation coefficient *R* ^2^. The error between paired variables was evaluated by the Bland Altman plot. The linear regression was determined using the least squares method. The results were presented in the form of mean ± standard deviation (SD).

**Figure 2:**
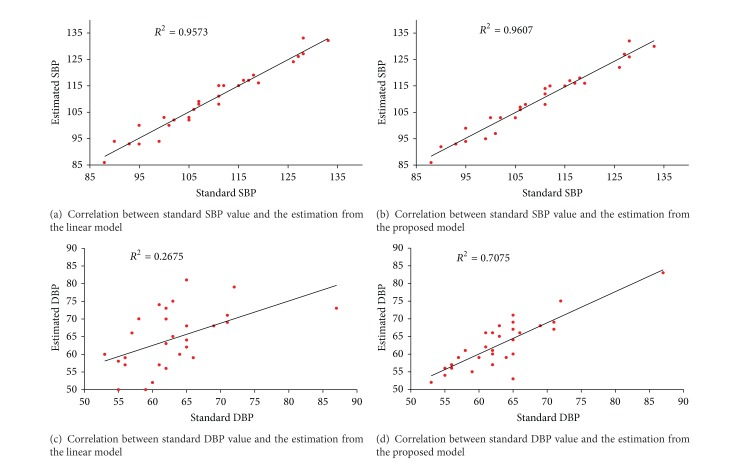
Correlation between the estimated blood pressure and the standard blood pressure for both linear model and the proposed model.

The blood pressure measured by the oscillometry was regarded as the standard value. Correlation between estimated blood pressure values from the two estimation models and the standard value was first analyzed, as shown in Figure 2. It appears that both estimation models could provide an acceptable estimation on SBP as the correlation coefficient *R* ^2^ around 0.96 (*P* < 0.001) for the estimation results derived from two models. However, for the DBP, the proposed model showed a better performance with *R* ^2^ of 0.71 (*P* < 0.001), while it is 0.27 (*P* < 0.01) for the linear model estimation results. The correlation analysis indicates that the proposed method provides better estimation performance on blood pressure especially for the DBP.

**Figure 3:**
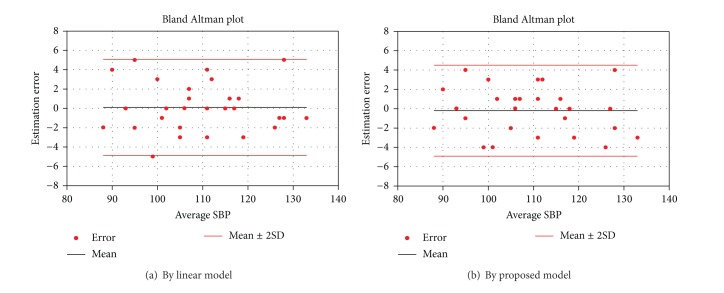
Bland Altman plot of estimation error of systolic blood pressure.

**Figure 4:**
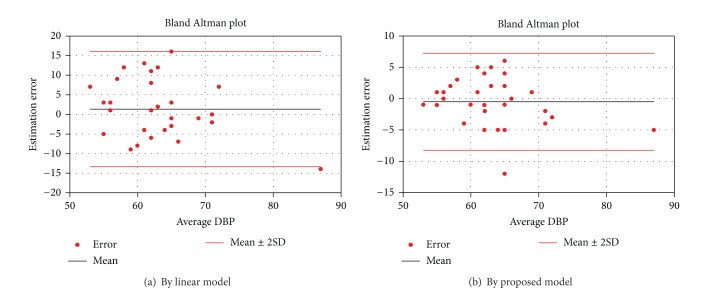
Bland Altman plot of estimation error of diastolic blood pressure.

The mean estimation error of the proposed model was −0.2 ± 2.4 mmHg and −0.5 ± 3.9 mmHg, while it was 0.1 ± 2.5 mmHg and 1.3 ± 7.4 mmHg from the linear model, for the SBP and DBP, respectively. The result again showed that the proposed method had a better performance on the DBP estimation. This is evident in the Bland Altman plot shown in Figures 3 and 4.

## 4. Discussions

As an important risk factor, blood pressure has prognostic value for stroke. Continuous blood pressure measurement will assist for the preventing and predicting of stroke. The PTT-based blood pressure estimation provides the most practical solution for the continuous measurement since the required signal (ECG and PPG) can be obtained by wearable devices. The purpose of this study was to develop a PTT-based blood pressure estimation method with personalized model and easy implementation. The underlying mechanism blood pressure estimation is that pulse wave velocity, which is the inverse of PTT, is directly determined by the elasticity of vessel wall that is associated with blood pressure level [43]. Wong et al. [44] carried out a longitudinal study to show that PTT-based blood pressure estimation had a good performance within half year but the estimation accuracy went worse for longer time. Payne et al. [30] proved that pulse wave velocity was deeply related to SBP but the estimation of DBP was still barely satisfactory. Factors that contribute to blood pressure regulation were suggested to be included in the PTT-based blood pressure estimation model [18, 45]. Baek et al. [46] tried to take heart rate and arterial stiffness into account for the blood pressure estimation model, which improved the estimation accuracy. However, the multifactor model is too complex for application.

Considering all above factors, we proposed a PTT-based blood pressure estimation method including a new model with compensation and calibration procedure. Hydrostatic effect has been approved to be influencing blood pressure level and included in current method in calibration to derive personalized coefficients in the estimation model for each individual [35, 47]. Sitting and standing postures were adopted as the calibration procedure in current study. The results showed that estimation from linear model with calibration already can provide a good performance for SBP estimation, indicating that personalized model is important for blood pressure estimation. However, the estimation of the DBP by the linear model with calibration was still poor, which was consistent with previous reports [30].

It is well known that the variability in heart rate and blood pressure carries important neural control information for the cardiovascular system [39–41]. One previous study showed that the variability in PTT had high coherence with that in blood pressure, indicating that some regulating factors affect both signals, simultaneously. Therefore, in current study, the variation in PTT was included as one compensation part to reflect the blood pressure change, while the variability in PTT was adopted as the other compensation part to indicate the neural control. The results made it evident that the model with the compensation provided a better estimation, especially for the DBP. It is possibly because the regulation mechanism of DBP has been included in the estimation model by employing the compensation. As a whole, the model with compensation can provide a better blood pressure estimation. Variability in PTT has potential to improve the blood pressure estimation.

The proposed model has shown promising potential for continuously monitoring blood pressure. As the risk of stroke is much dependent on the blood pressure level [5–7], such a method would help blood pressure control and enhance the hypertension management, especially for stroke patients. It will be much helpful if such a method can be introduced in the stroke rehabilitation to achieve a better blood pressure control. Patients must benefit from the hypertension management for preventing stroke or predicting stroke.

Although the current study shows promising results, there is one limitation. This study only tested the proposed method on healthy young subjects. It is well known that in hypertension patients, the blood pressure regulation is different from the healthy. Such method needs to be validated further on people with different conditions, such as patients with stroke and hypertension.

## Figures and Tables

**Table 1:** Procedure of routine data collection.

Tasks	Duration	Remarks
Keep the testing posture	2 mins	
ECG and PPG recording	3 mins	
Blood pressure measurement		
Rest	5 mins	
Blood pressure measurement		
Rest	5 mins	Optional
Blood pressure measurement		

**Table 2:** Outline of the experiment.

Pretest	10-minute rest
	Preparation

Calibration-sitting	ECG and PPG recording
	Blood pressure measurement

Calibration-standing	2-minute posture holding
	ECG and PPG recording
	Blood pressure measurement

Estimation-test (sitting)	2-minute posture holding
	ECG and PPG recording
	Blood pressure measurement
